# Lead in Cocoa Products: Where Does Contamination Come From?

**Published:** 2005-10

**Authors:** David A. Taylor

Manufactured cocoa products frequently have higher lead concentrations than other foods, even though cocoa beans, the main ingredient, have some of the lowest reported lead levels for any natural food. In 2001 the Codex Alimentarius Commission, an international body based in Rome, proposed reducing the maximum permissible level of lead in cocoa products by half, to 100 nanograms per gram (ng/g) for cocoa butter and 1,000 ng/g for cocoa powder. At a March 2002 meeting in West Africa, where most of the world’s cocoa supply originates, producers agreed that to reduce lead in their products, they needed research to identify the source of contamination. Now a U.S.–Nigerian research team has uncovered some of the first clues about where the lead is coming from **[*EHP* 113:1344–1348]**.

Lead contamination of candies has been recognized as a problem since 1820, when a British study found the poison widespread in London confectionary products. In recent years, documented lead content in candy has ranged from a mean concentration of 21 ng/g in milk chocolate bars in an Australian study to an average of 1,920 ng/g in chocolates seen in research in India. In Nigeria, a 1999 study found an average of 310 ng/g lead in cocoa powders. (For comparison, the mean U.S. lead concentration for apples is 20 ng/g, 200 ng/g for dry table wine, and 100 ng/g for canned pineapple.) Lead is known to cause anemia, muscle weakness, and brain damage, with children particularly susceptible to effects.

In the current study, the researchers studied the lead isotopic compositions of cocoa beans and shells from six farms in Nigeria’s top three producing states to determine if soil or farm sources might be the cause of lead contamination. The team took bean and sediment samples and homogenized them to make composites for soil, beans, and cocoa bean shells for each farm. They analyzed lead concentrations using high-resolution inductively coupled plasma mass spectrometry to make preliminary isotopic measurements, followed by thermal ionization mass spectrometry measurements.

The lead concentrations for cocoa beans ranged from less than 0.103 to 1.78 ng/g, averaging 0.512 ng/g—among the lowest lead concentrations reported for any food. The average concentrations found in the cocoa bean shells, however, was about 320-fold higher (160 ng/g). Soils showed a range of isotopic compositions overlapping those of the shells.

The cocoa bean shells all had an extremely similar isotopic composition, indicating a singular source of contamination, perhaps leaded gasoline. The authors conclude that although the soil may have caused a small degree of the contamination, the narrower range of isotopic composition in the shells suggested the more singular source of contamination was the true culprit. According to the paper, cocoa bean shells are known to be very effective at removing lead from solutions. So, although they provide excellent protection of the bean inside, the shells may also serve to contaminate the cocoa beans during fermentation or drying.

The team also compared the cocoa beans with finished cocoa products and found much higher lead concentrations and greater variability in the isotopic composition among the finished products. They therefore deduced that most of the contamination occurred after the cocoa left the farm stage.

The researchers conclude that while cocoa bean shells may be one source of lead, most contamination occurs during shipping or processing of the beans and in manufacturing. Further research on those stages of the process will help to isolate the source.

## Figures and Tables

**Figure:**
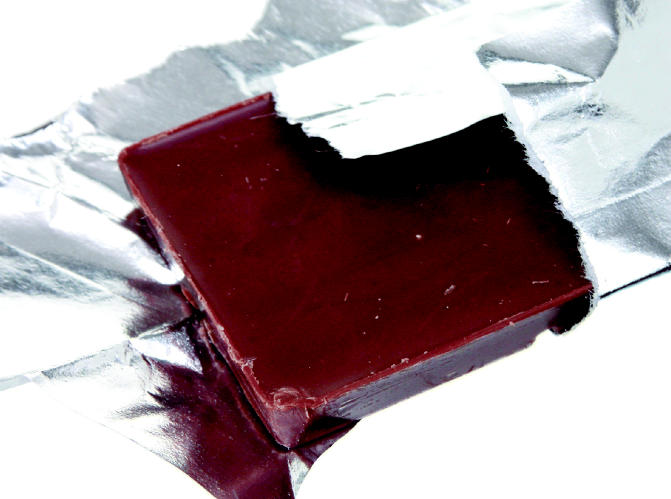
Searching for the golden ticket. Cocoa beans are naturally low in lead, but cocoa products frequently are not. Now researchers are following new clues to identify the source of the contamination.

